# Survival and factors associated with intensive care unit mortality among adults with impaired consciousness in Benin, a low-resource setting: A retrospective cohort study

**DOI:** 10.1371/journal.pone.0350120

**Published:** 2026-06-24

**Authors:** Mahunan Gerard Sossou, Dieu donne Gnonlonfoun

**Affiliations:** 1 Doctoral School of Health Sciences, Université d’Abomey-Calavi, Cotonou, Benin; 2 School of Public Health, Université libre de Bruxelles, Brussels, Belgium; 3 Faculty of Health Sciences, Laboratory of Epidemiology of Chronic and Neurological Diseases, Université d’Abomey-Calavi, Cotonou, Benin; 4 Department of Neurology, Centre National Hospitalier Universitaire Hubert K. Maga, Cotonou, Benin; Universiti Sains Malaysia, MALAYSIA

## Abstract

**Introduction:**

Impaired consciousness is a common and potentially life-threatening condition that frequently requires admission to intensive care units (ICUs). In sub-Saharan Africa, data on outcomes among critically ill patients with impaired consciousness remain limited, particularly in resource-limited settings. This study aimed to estimate survival and identify factors associated with ICU mortality among adults with impaired consciousness admitted to the ICU of the national referral hospital in Benin.

**Methods:**

This single-center retrospective cohort study used secondary data from the ICU database covering January 2015 to June 2017. Survival was estimated using the Kaplan–Meier method, and factors associated with ICU mortality were identified using Cox proportional hazards models.

**Results:**

Among 416 patients, 279 died, yielding an ICU mortality of 67.1%. The median time to death in the ICU was 5 days. Survival probabilities declined from 77.6% on day 1 to 21.9% on day 15 and 13.7% on day 30. In multivariable analysis, older age and lower Glasgow Coma Scale score showed time-varying associations with ICU mortality. Lower systolic blood pressure, higher body temperature, absence of traumatic brain injury, and absence of oxygen therapy were independently associated with higher ICU mortality.

**Conclusion:**

ICU mortality among adults with impaired consciousness admitted to this ICU was very high. This observation is consistent with constraints in essential critical care resources. Simple clinical parameters available at admission may support early risk stratification. Strengthening essential critical care components, particularly basic physiological monitoring and reliable oxygen supply, warrants consideration in sub-Saharan African ICUs.

## Introduction

Impaired consciousness is a common and potentially life-threatening clinical condition that frequently requires admission to emergency departments (EDs) or intensive care units (ICUs). Approximately 4% to 10% of ED patients present with impaired consciousness, and many require ICU admission due to illness severity or the need for close monitoring and supportive care [1,2].

In sub-Saharan Africa, evidence on the epidemiology and outcomes of impaired consciousness in critically ill patients remains limited. This gap is particularly relevant in low- and middle-income countries (LMICs), where ICU capacity is often constrained and ICU mortality among critically ill patients remains high [3–5]. Context-specific prognostic data are therefore essential for triage and early clinical decision-making in resource-limited settings. A White Paper jointly supported by the International Federation for Emergency Medicine and the World Federation of Intensive and Critical Care Medicine has also highlighted this need [6].

In Benin, to our knowledge, no study has specifically examined survival outcomes among adults with impaired consciousness admitted to the ICU. A better understanding of factors associated with ICU mortality is needed to guide triage, early management, and resource allocation. This study therefore aimed to estimate survival and identify factors associated with ICU mortality among adults with impaired consciousness admitted to the ICU of the national referral hospital in Benin.

## Methods

### Study design

This single-center retrospective cohort study used secondary data from an existing ICU database covering January 2015 to June 2017. The study is reported in accordance with the Strengthening the Reporting of Observational Studies in Epidemiology (STROBE) guidelines for observational studies [7]. The completed STROBE checklist is provided in S1 Checklist in S1 File.

### Setting

The study was conducted in the Clinique Universitaire Polyvalente d’Anesthésie et de Réanimation (CUPAR), the ICU of the Centre National Hospitalier Universitaire Hubert K. Maga (CNHU-HKM), the national referral hospital in Cotonou, Benin. CNHU-HKM is part of Benin’s university hospital network and hosts the largest ICU in the country. CUPAR has approximately 20 beds organized around a central monitoring area and is staffed by anesthesiologist–intensivists, residents, senior technicians, and specialized nurses. The unit is equipped with a central oxygen–air–vacuum supply system, mechanical ventilators, patient monitoring devices, and standard resuscitation equipment. CUPAR admits critically ill patients from other hospital departments and from external healthcare facilities, the latter primarily through the emergency department. Admission is based on clinical severity and the need for close monitoring and organ support, and is not restricted by the level of impaired consciousness.

### Participants

The study included all consecutive patients aged 18 years and older with a Glasgow Coma Scale (GCS) score of 3–14 at ICU admission between January 2015 and June 2017, capturing the full spectrum of impaired consciousness encountered in routine practice at the ICU (CUPAR). Patients younger than 18 years were excluded. The level of consciousness was assessed by the medical team using the GCS [8], as detailed in S2 Appendix in S1 File. Patients were followed until ICU death or discharge. No a priori sample size or power calculation was performed.

### Variables

The outcome of interest was ICU death among patients with impaired consciousness. Death was defined clinically as the cessation of all cardiorespiratory activity, with bilaterally fixed and dilated pupils. ICU length of stay was calculated as the number of days from ICU admission to ICU death or discharge alive, with discharge alive treated as a censoring event. No post-discharge follow-up data were available.

Independent variables were selected based on prior literature, clinical relevance in critical care, and reported associations with ICU mortality among adults with impaired consciousness. Variables were grouped into four domains: sociodemographic characteristics (age [18–59 years; ≥ 60 years] and sex [female; male], with age categorized according to the World Health Organization [WHO] definition of older age [9]); comorbidity- and lifestyle-related factors (hypertension, diabetes, traumatic brain injury [TBI], prior surgery, alcohol consumption, and tobacco use); clinical characteristics at admission (admission delay, body temperature, systolic and diastolic blood pressure, oxygen saturation, Glasgow Coma Scale [GCS] score, and cause of impaired consciousness); and management-related variables (orotracheal intubation, oxygen therapy, mechanical ventilation, dialysis, sedation, and blood transfusion) [10–19]. Detailed descriptions, coding schemes, and variable categorizations are provided in S3 Appendix in S1 File.

### Data sources

Data for this study were accessed in February 2025 from an existing fully anonymized ICU database of CNHU-HKM, which had been retrospectively constructed in 2019 from archived medical records of patients discharged from, or deceased in, the ICU between January 2015 and June 2017. During the original database construction, data abstraction was performed by trained staff using pretested data collection forms, and the collected information was entered into Epi Info version 7.2.2.16. The present analysis was conducted exclusively on the anonymized dataset provided to the investigators.

### Data cleaning and processing

Data were exported to Microsoft Excel and processed using R, version 4.5.2. The outcome had no missing values. Several independent variables contained missing data: age (n = 4), body temperature (n = 17), systolic blood pressure (n = 28), diastolic blood pressure (n = 28), oxygen saturation (n = 3), and GCS score (n = 36). Observations with missing key inclusion variables, particularly age or GCS score, were excluded.

The overall proportion of missing data in the initial study sample was low (2.5%), with one predominant pattern (8 observations) and three isolated patterns (one observation each). Analyses were restricted to complete cases. Baseline characteristics of the initial and final analytic samples did not differ significantly, as shown in S4 Table in S1 File.

### Statistical analysis

Categorical variables are presented as frequencies and percentages, and continuous variables as medians and interquartile ranges (IQR), as most continuous variables were non-normally distributed, assessed by Q–Q plots. ICU mortality was estimated as a proportion, and survival over the ICU stay was analyzed using Kaplan–Meier curves; the median time to death in the ICU was reported with 95% confidence intervals (95% CI).

Factors associated with ICU mortality were identified using a Cox proportional hazards model, with ICU length of stay as the time scale; patients discharged alive were treated as censored observations. Independent variables with p ≤ 0.20 in univariable analyses or with established relevance to critical care were considered for inclusion in the multivariable model. Multicollinearity was assessed using the variance inflation factor (VIF), and the Akaike information criterion (AIC) guided variable selection when correlations were identified.

Proportional hazards assumptions were assessed using Schoenfeld residuals. Violations were addressed by modeling time-varying effects using the tt() function with a log-time interaction, or by stratification as appropriate. Time-varying effects were specified for age, diastolic blood pressure, and GCS score. Orotracheal intubation and mechanical ventilation were included as stratification variables. Significant collinearity was observed between traumatic brain injury and cause of impaired consciousness (VIF > 5); the model including traumatic brain injury was retained based on a lower AIC.

Adjusted hazard ratios (aHRs) with 95% confidence intervals (CIs) were estimated. Model stability was assessed using bootstrap resampling with 1,000 iterations. A sensitivity analysis excluding patients with traumatic brain injury was performed to assess the robustness of the findings. Subgroup analyses stratified by Glasgow Coma Scale score (≤ 8 vs > 8) were also conducted. All analyses were performed using R (version 4.5.2).

### Ethical considerations

This study used secondary data from the ICU database of CNHU-HKM. The protocol for the initial creation of the database from archived medical records was reviewed and approved by the Medical Statistics and Research Department (Service de la statistique médicale et de la recherche) of CNHU-HKM, Cotonou, Benin (Ref. No. 1759/MS/CNHU-HKM/PCME/SSMR). The dataset was fully anonymized prior to access by the investigators, and no identifiable information was available at any stage of the study. The requirement for informed consent was waived by the approving body due to the retrospective nature of the study and the use of anonymized data.

## Results

**Fig 1:**
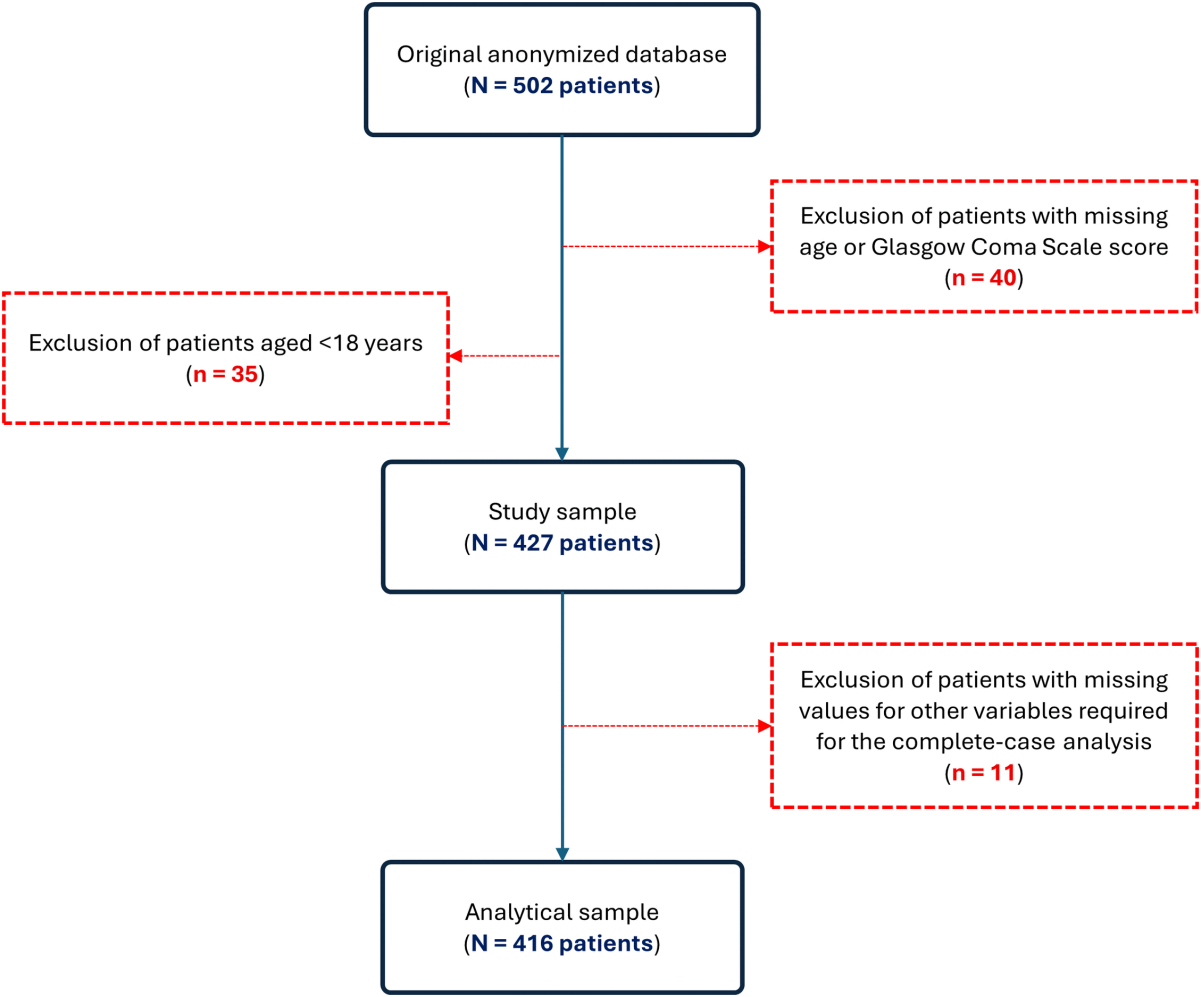
Flow diagram showing the selection process of study participants.

The original anonymized database included 502 patients. Patients with missing age or Glasgow Coma Scale (GCS) score were excluded (n = 40), followed by exclusion of patients aged <18 years (n = 35), yielding a study sample of 427 patients. An additional 11 patients with missing values for other variables required for the complete-case analysis were excluded, resulting in a final analytical sample of 416 patients (Fig 1).

### General characteristics

Table 1 summarizes the general characteristics of the study participants. The median age was 45.0 years (IQR 30.0–60.0), and the sex ratio was 0.94 (202 men, 214 women). The median follow-up time, corresponding to ICU length of stay, was 3.0 days (IQR 1.0–7.0), with a maximum ICU stay of 65 days. Among the 416 adults with impaired consciousness, 279 deaths occurred in the ICU, resulting in an ICU mortality of 67.1% (95% CI 62.5–71.6). Vascular (45.2%), metabolic (14.7%), and infectious (9.9%) causes were the leading causes of non-traumatic impaired consciousness, while traumatic causes represented 19.0%.

**Table 1:** General characteristics of the study participants.

	Categories	Descriptive statistics
N		416
***Sociodemographic variables***		
Age (years), median (IQR)		45.0 (30.0–60.0)
Age categories, n (%)	18–59 years	304 (73.1)
	≥ 60 years	112 (26.9)
Sex, n (%)	Female	214 (51.4)
	Male	202 (48.6)
***Comorbidity- and lifestyle-related variables***		
Hypertension, n (%)	Yes	165 (39.7)
	No	251 (60.3)
Diabetes, n (%)	Yes	59 (14.2)
	No	357 (85.8)
TBI, n (%)	Yes	84 (20.2)
	No	332 (79.8)
Prior surgery, n (%)	Yes	108 (26.0)
	No	308 (74.0)
Alcohol consumption, n (%)	Yes	146 (35.1)
	No	270 (64.9)
Tobacco use, n (%)	Yes	12 (2.9)
	No	404 (97.1)
***Clinical variables at admission***		
Admission delay (days), median (IQR)		1.0 (0–3.0)
Admission delay categories, n (%)	< 1 day	171 (41.1)
	≥ 1 day	245 (58.9)
Body temperature (°C), median (IQR)		37.6 (37.0–38.5)
Body temperature categories, n (%)	Normal temperature	229 (55.0)
	Hypothermia	11 (2.6)
	Hyperthermia	176 (42.3)
SBP (mmHg), median (IQR)		135.5 (111.8–164.2)
SBP categories, n (%)	Normal SBP	186 (44.7)
	SBP ≤ 90 (Hypotension)	39 (9.4)
	SBP ≥ 140 (Hypertension)	191 (45.9)
DBP (mmHg), median (IQR)		82.0 (64.8–100.0)
DBP categories, n (%)	Normal DBP	204 (49.0)
	DBP ≤ 50 (Hypotension)	49 (11.8)
	DBP ≥ 90 (Hypertension)	163 (39.2)
Oxygen saturation (%), median (IQR)		99.0 (96.8–100)
Oxygen saturation categories, n (%)	Normal saturation	347 (83.4)
	Desaturation (SaO₂ < 95%)	69 (16.6)
Glasgow Coma Scale score, median (IQR)		8.0 (6.0–10.0)
Glasgow Coma Scale score categories, n (%)	Mild impairment (GCS score 14–13)	22 (5.3)
	Moderate impairment (GCS score 12–9)	148 (35.6)
	Severe impairment/Coma (GCS score 8–3)	246 (59.1)
Cause of impaired consciousness, n (%)	Vascular	188 (45.2)
	Metabolic	61 (14.7)
	Infectious	41 (9.9)
	Other (anoxic brain injury, electrical injury, etc.)	13 (3.1)
	Tumor-related	12 (2.9)
	Toxic/Drug-related	11 (2.6)
	Undetermined	10 (2.4)
	Seizure-related	1 (0.2)
	Traumatic	79 (19.0)
***Management-related variables***		
Orotracheal intubation, n (%)	Yes	295 (70.9)
	No	121 (29.1)
Oxygen therapy, n (%)	Yes	248 (59.6)
	No	168 (40.4)
Mechanical ventilation, n (%)	Yes	253 (60.8)
	No	163 (39.2)
Dialysis, n (%)	Yes	5 (1.2)
	No	411 (98.8)
Sedation, n (%)	Yes	137 (32.9)
	No	279 (67.1)
Blood transfusion, n (%)	Yes	54 (13.0)
	No	362 (87.0)
***Time variable***		
ICU length of stay (days), median (IQR)		3.0 (1.0–7.0)

TBI, traumatic brain injury; SBP, systolic blood pressure; DBP, diastolic blood pressure; GCS, Glasgow Coma Scale.

n (%), frequency (percentage); median (IQR), median (interquartile range).

### Survival analysis

**Fig 2:**
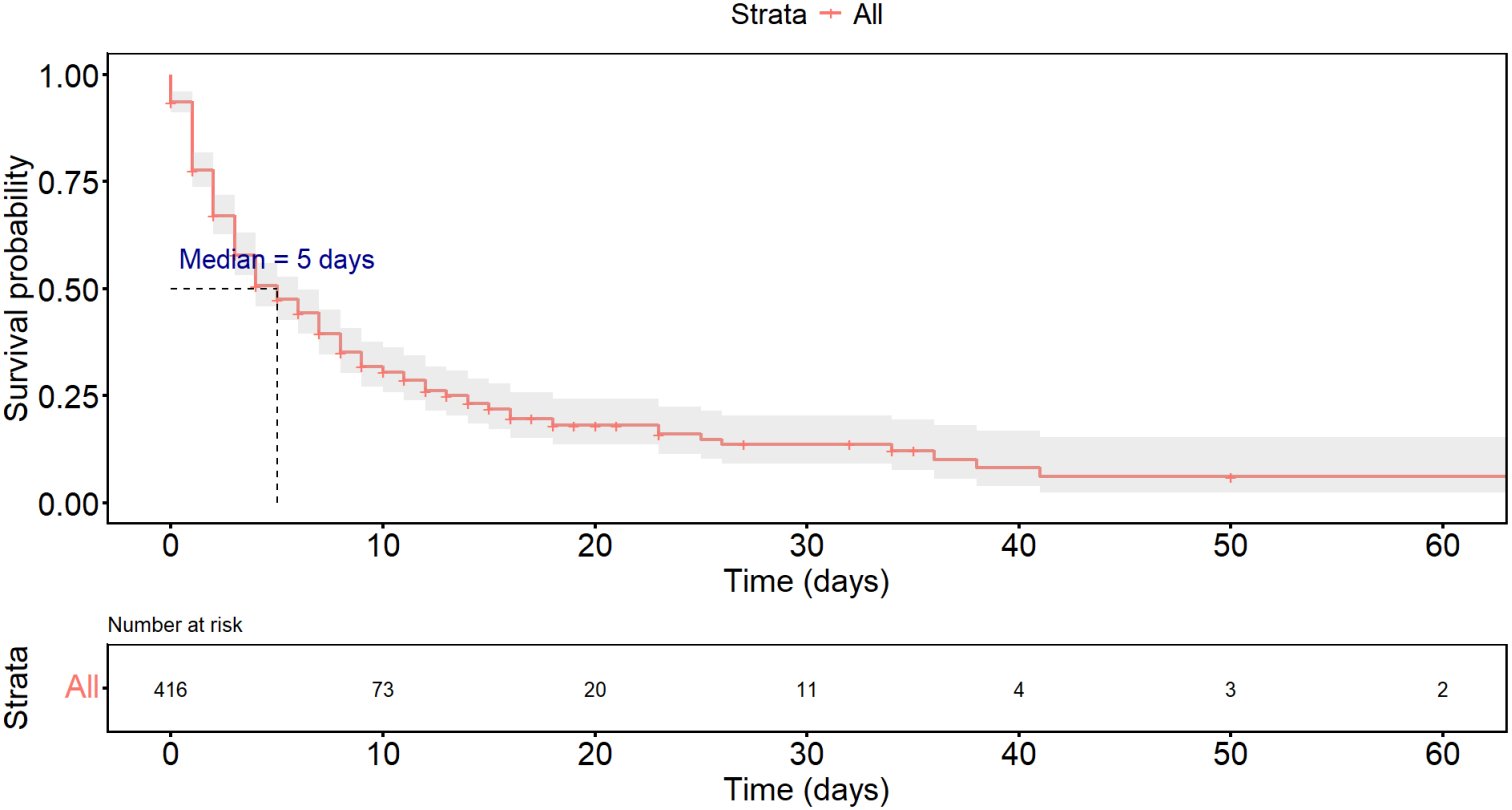
Kaplan–Meier survival curve for adults with impaired consciousness admitted to the ICU.

Kaplan–Meier analysis estimated a median time to death in the ICU of 5 days (95% CI 4–6) (Fig 2). The estimated survival probabilities were 77.6% (95% CI 73.7–81.7) on day 1, 21.9% (95% CI 17.2–27.8) on day 15, and 13.7% (95% CI 9.2–20.4) on day 30.

### Factors associated with ICU mortality

Variables with a p-value ≤ 0.20 in univariable analysis were considered for inclusion in the multivariable Cox proportional hazards model. These variables were age, hypertension, traumatic brain injury, body temperature, systolic and diastolic blood pressure, oxygen saturation, Glasgow Coma Scale score, cause of impaired consciousness, orotracheal intubation, oxygen therapy, mechanical ventilation, and blood transfusion. Admission delay was also included because of its clinical relevance in critical care. Univariable associations with ICU mortality are presented in Table 2.

**Table 2:** Factors associated with ICU mortality among adults with impaired consciousness (univariable Cox proportional hazards models).

	Categories	HR	95% CI	p-value
***Sociodemographic variables***				
Age (years)		1.01	[1.00–1.02]	0.001^a^
Sex	Female	1*		
	Male	1.03	[0.81–1.30]	0.806
***Comorbidity- and lifestyle-related variables***				
Hypertension	No	1*		
	Yes	1.27	[1.01–1.61]	0.045^a^
Diabetes	No	1*		
	Yes	1.11	[0.80–1.54]	0.550
TBI	No	1*		
	Yes	0.61	[0.45–0.83]	0.002^a^
Prior surgery	No	1*		
	Yes	0.94	[0.70–1.25]	0.658
Alcohol consumption	No	1*		
	Yes	0.98	[0.77–1.25]	0.860
Tobacco use	No	1*		
	Yes	0.90	[0.43–1.92]	0.790
***Clinical variables at admission***				
Admission delay (days)		1.00	[0.97–1.04]	0.822^b^
Body temperature (°C)		1.22	[1.10–1.36]	<0.001^a^
SBP (mmHg)		0.99	[0.99–1.00]	<0.001^a^
DBP (mmHg)		0.99	[0.98–0.99]	<0.001^a^
Oxygen saturation (%)		0.95	[0.93–0.97]	<0.001^a^
Glasgow Coma Scale score		0.80	[0.76–0.84]	<0.001^a^
Cause of impaired consciousness	Non-traumatic	1*		
	Traumatic	0.61	[0.45–0.84]	0.002^a^
***Management-related variables***				
Orotracheal intubation	No	1*		
	Yes	3.21	[2.25–4.58]	<0.001^a^
Oxygen therapy	No	1*		
	Yes	0.43	[0.34–0.55]	<0.001^a^
Mechanical ventilation	No	1*		
	Yes	2.22	[1.67–2.95]	<0.001^a^
Dialysis	No	1*		
	Yes	1.17	[0.44–3.14]	0.756
Sedation	No	1*		
	Yes	1.05	[0.82–1.33]	0.716
Blood transfusion	No	1*		
	Yes	0.61	[0.43–0.88]	0.008^a^

TBI, traumatic brain injury; SBP, systolic blood pressure; DBP, diastolic blood pressure.

* Reference category.

^a^Significant at the 0.20 level.

^b^Included based on relevance to critical care.

**Fig 3:**
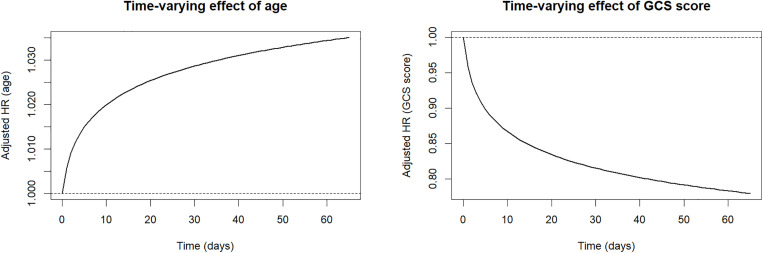
Time-varying adjusted hazard ratios for age and Glasgow Coma Scale score among adults with impaired consciousness.

In the multivariable Cox proportional hazards model, age and Glasgow Coma Scale (GCS) score showed time-varying associations with ICU mortality. The direction of the association varied over time: increasing with advancing age and decreasing with higher GCS scores (Fig 3).

Among other covariates, systolic blood pressure (aHR = 0.99; 95% CI 0.99–1.00; p = 0.003), body temperature (aHR = 1.12; 95% CI 1.01–1.24; p = 0.031), oxygen therapy (aHR = 0.57; 95% CI 0.43–0.75; p < 0.001), and traumatic brain injury (aHR = 0.68; 95% CI 0.49–0.95; p = 0.025) were independently associated with ICU mortality (Table 3).

**Table 3:** Factors associated with ICU mortality among adults with impaired consciousness (multivariable Cox proportional hazards models, including standard and bootstrap-resampled estimates).

		Standard			Bootstrap		Effect type
		aHR	95% CI	p-value	aHR	95% CI	
***Time-varying effects***							
**Age** (years)		–	–	**0.002** ^**a**^	–	–	Time-varying effect
DBP (mmHg)		–	–	0.661	–	–	Time-varying effect
**Glasgow Coma Scale score**		–	–	**0.001** ^**a**^	–	–	Time-varying effect
***Fixed effects***							
Hypertension	No	1*			1*		
	Yes	0.90	[0.67–1.21]	0.497	0.89	[0.67–1.18]	Fixed effect
**TBI**	No	1*			1*		
	**Yes**	**0.68**	**[0.49–0.95]**	**0.025** ^**a**^	0.67	[0.47–0.95]	Fixed effect
Admission delay (days)		1.00	[0.96–1.04]	0.941	1.00	[0.96–1.03]	Fixed effect
**Body temperature** (°C)		**1.12**	**[1.01–1.24]**	**0.031** ^**a**^	1.12	[1.00–1.28]	Fixed effect
**SBP** (mmHg)		**0.99**	**[0.99–1.00]**	**0.003** ^**a**^	0.99	[0.99–1.00]	Fixed effect
Oxygen saturation (%)		0.98	[0.96–1.00]	0.050	0.98	[0.95–1.00]	Fixed effect
**Oxygen therapy**	No	1*			1*		
	**Yes**	**0.57**	**[0.43–0.75]**	**<0.001** ^**a**^	0.57	[0.41–0.78]	Fixed effect
Blood transfusion	No	1*			1*		
	Yes	0.67	[0.46–0.99]	0.041	0.67	[0.42–1.04]	Fixed effect
***Stratified variables***							
Orotracheal intubation		–	–	–	–	–	Stratification
Mechanical ventilation		–	–	–	–	–	Stratification

DBP, diastolic blood pressure; TBI, traumatic brain injury; SBP, systolic blood pressure.

* Reference category.

^a^Significant at the 0.05 level.

Bootstrap resampling confirmed the stability of these associations. In the internally validated final model, systolic blood pressure (aHR = 0.99; 95% CI 0.99–1.00), oxygen therapy (aHR = 0.57; 95% CI 0.41–0.78), and traumatic brain injury (aHR = 0.67; 95% CI 0.47–0.95) were independently associated with ICU mortality, whereas body temperature (aHR = 1.12; 95% CI 1.00–1.28) was not (Table 3).

In the sensitivity analysis excluding patients with traumatic brain injury, the associations of Glasgow Coma Scale score (time-varying), systolic blood pressure, and oxygen therapy with ICU mortality remained statistically significant. In contrast, the time-varying association of age and the association with body temperature were no longer statistically significant compared with the full cohort, as shown in S5 Table in S1 File.

In subgroup analyses stratified by Glasgow Coma Scale score (≤ 8 vs > 8), the associations of systolic blood pressure and oxygen therapy with ICU mortality remained statistically significant in the GCS score ≤ 8 subgroup. In contrast, the time-varying association of age and the associations of traumatic brain injury and body temperature with ICU mortality were no longer statistically significant in this subgroup compared with the full cohort, as shown in S6 Table in S1 File.

## Discussion

This study estimated survival and identified factors associated with ICU mortality among adults with impaired consciousness admitted to the ICU of the national referral hospital in Benin. The findings showed a very high ICU mortality (67.1%), with a median time to death in the ICU of 5 days. Age and Glasgow Coma Scale (GCS) score showed time-varying associations with ICU mortality, whereas systolic blood pressure, body temperature, oxygen therapy, and traumatic brain injury were independently associated with the outcome.

These findings should be interpreted in the context of the limited critical care capacity typical of many low- and middle-income countries during the study period. Although the data are based on ICU admissions from 2015 to 2017, they remain an important source of information on ICU outcomes among adults with impaired consciousness in Benin. This study therefore contributes valuable evidence in a still under-described clinical setting.

Across sub-Saharan Africa, the ICU mortality observed in this study (67.1%) was substantially higher than that reported in many countries, including the Democratic Republic of Congo (35.2%), Ethiopia (35%), and Nigeria (52.2%) [10,12,13,19,20], but lower than the 82.9% reported in a stroke-specific cohort in Senegal [21]. These differences are consistent with variations in case mix, severity at admission, and underlying health system capacity. In this study, the short median time to death in the ICU (5 days) indicates that most deaths occurred early during ICU stay. This pattern may reflect the absence of neurocritical care, limited life-support resources, and constraints in the intensity and continuity of early critical care delivery in this resource-limited setting. The absence of an association between admission delay and ICU mortality further suggests that the observed mortality pattern is not explained by delayed ICU admission alone.

Consistent with prior studies [10,12,17], older age and lower Glasgow Coma Scale (GCS) score were associated with higher ICU mortality. The time-varying association of age is compatible with declining physiological resilience with increasing age. Each additional point in the GCS, indicating a higher level of consciousness, was associated with a reduced hazard of ICU death, although the protective association diminished over time. Systolic blood pressure (SBP) and body temperature at admission were also associated with ICU mortality. When expressed per 10 mmHg increase, SBP was associated with a lower hazard of ICU death (aHR = 0.90; 95% CI 0.90–1.00). Lower SBP (hypotension) may compromise cerebral perfusion [16], whereas higher body temperature (hyperthermia) can exacerbate neuronal injury [22]. However, the association of body temperature was not confirmed after bootstrap resampling, and should therefore be interpreted with caution. In resource-limited ICUs, active monitoring and management of secondary systemic insults may be a feasible approach to improving outcomes.

Receipt of oxygen therapy was associated with lower ICU mortality; however, this association should be interpreted cautiously. In this retrospective study, oxygen therapy may have been more frequently administered to patients with less severe illness or better physiological reserve, and confounding by indication cannot be excluded in the absence of severity scores such as APACHE II or SOFA. This finding is nonetheless consistent with the clinical importance of preventing hypoxemia in critically ill patients with impaired consciousness [23]. Interestingly, patients with traumatic brain injury (TBI) had lower ICU mortality. This finding is consistent with case mix differences: the TBI subgroup may include younger patients with isolated or less severe injuries and fewer comorbidities, whereas non-traumatic impaired consciousness is more often associated with diffuse or irreversible brain injury [14].

Blood transfusion was associated with lower ICU mortality in the standard model (aHR = 0.67; 95% CI 0.46–0.99; p = 0.041), but this was not confirmed after bootstrap resampling (95% CI 0.42–1.04). Given the small number of transfused patients (n = 54) and the possibility of confounding by indication, this finding should be interpreted with caution.

The sensitivity analysis excluding patients with traumatic brain injury supported the robustness of the associations observed for Glasgow Coma Scale score (time-varying), systolic blood pressure, and oxygen therapy. In contrast, the time-varying association of age and the association with body temperature were less stable, suggesting that they should be interpreted with caution.

The subgroup analyses suggest that the associations of systolic blood pressure and oxygen therapy with ICU mortality were more consistent among patients with GCS score ≤ 8, whereas the associations of age (time-varying), traumatic brain injury, and body temperature appeared less stable in this subgroup. However, given the reduced sample size, these findings should be interpreted with caution.

Strengths of this study include a clinically relevant ICU setting, validated neurological assessment using the GCS, a relatively large sample size (n = 416), minimal missing data, and robust statistical analyses, including Cox proportional hazards models, bootstrap resampling, sensitivity analyses, and subgroup analyses. Adjustment for key confounders further enhanced internal validity.

Limitations include the absence of important ICU variables, such as duration of mechanical ventilation, vasopressor use, and severity scores (APACHE II or SOFA). Some management-related variables included in the model may have introduced confounding by indication. In this low-resource context, residual confounding from unmeasured variables, such as pre-ICU care, socioeconomic status, and treatment adherence, as well as potential heterogeneity in ICU practices (sedation and orotracheal intubation protocols), may have influenced outcomes. Follow-up was limited to ICU stay, which may have underestimated overall in-hospital mortality. Finally, the single-center design, inclusion of only ICU patients, and exclusion of pediatric patients may limit the generalizability of the findings.

## Conclusions

ICU mortality among adults with impaired consciousness admitted to the ICU in Benin remains very high, with most deaths occurring early during the ICU stay. Several simple clinical parameters available at admission were associated with ICU mortality, supporting their potential use for early risk stratification. These findings highlight important gaps in essential ICU care in low-resource settings. Strengthening basic critical care capacity, particularly physiological monitoring, early supportive management, and reliable oxygen supply, along with the development of context-adapted ICU guidelines and national critical care registries, may be relevant priorities for improving care in sub-Saharan African ICUs. Further multicenter and prospective studies are needed to inform scalable strategies for strengthening critical care systems in the region.

## Supporting information

S1 File Supporting information. This file contains all supplementary materials (S1 Checklist, S2 Appendix, S3 Appendix, S4 Table, S5 Table, S6 Table) referenced in the manuscript.
(PDF)
