# Supplementary material for: Survival and factors associated with intensive care unit mortality among adults with impaired consciousness in Benin, a low-resource setting: A retrospective cohort study
Source: PLoS One. 2026 Jun 24;21(6):e0350120. doi: 10.1371/journal.pone.0350120 (PMC13293454; doi:10.1371/journal.pone.0350120)
Supplement: S1 File — This file contains all supplementary materials (S1 Checklist, S2 Appendix, S3 Appendix, S4 Table, S5 Table, S6 Table) referenced in the manuscript. (PDF) [file pone.0350120.s001.pdf]

Supporting information

S1 Checklist. STROBE checklist.

	Item	Recommendation	Manuscript page
**Title and abstract**	1	(*a*) Indicate the study's design with a commonly used term in the title or the abstract	Pages 1 and 2
		(*b*) Provide in the abstract an informative and balanced summary of what was done and what was found	Pages 2-3
**Introduction**			
**Background/rationale**	2	Explain the scientific background and rationale for the investigation being reported	Pages 3-4
**Objectives**	3	State specific objectives, including any prespecified hypotheses	Page 4, lines 62-64
**Methods**			
**Study design**	4	Present key elements of study design early in the paper	Page 4
**Setting**	5	Describe the setting, locations, and relevant dates, including periods of recruitment, exposure, follow-up, and data collection	Pages 4-5
**Participants**	6	(*a*) *Cohort study*—Give the eligibility criteria, and the sources and methods of selection of participants. Describe methods of follow-up<br>*Case-control study*—Give the eligibility criteria, and the sources and methods of case ascertainment and control selection. Give the rationale for the choice of cases and controls<br>*Cross-sectional study*—Give the eligibility criteria, and the sources and methods of selection of participants	Page 5
		(*b*) *Cohort study*—For matched studies, give matching criteria and number of exposed and unexposed<br>*Case-control study*—For matched studies, give matching criteria and the number of controls per case	N/A
**Variables**	7	Clearly define all outcomes, exposures, predictors, potential confounders, and effect modifiers. Give diagnostic criteria, if applicable	Pages 5-6
**Data sources/ measurement**	8	For each variable of interest, give sources of data and details of methods of assessment	Page 6

		(measurement). Describe comparability of assessment methods if there is more than one group	
**Bias**	9	Describe any efforts to address potential sources of bias	Page 7, lines 139-141
**Study size**	10	Explain how the study size was arrived at	Pages 6-7, Data cleaning and processing
**Quantitative variables**	11	Explain how quantitative variables were handled in the analyses. If applicable, describe which groupings were chosen and why	Page 7, lines 132-134
**Statistical methods**	12	(*a*) Describe all statistical methods, including those used to control for confounding	Pages 7-8
		(*b*) Describe any methods used to examine subgroups and interactions	Page 8, lines 154-155
		(*c*) Explain how missing data were addressed	Page 7, lines 127-130
		(*d*) *Cohort study*—If applicable, explain how loss to follow-up was addressed<br>*Case-control study*—If applicable, explain how matching of cases and controls was addressed<br>*Cross-sectional study*—If applicable, describe analytical methods taking account of sampling strategy	Page 7, lines 138-139
		(*e*) Describe any sensitivity analyses	Page 8, lines 152-154
**Results**			
**Participants**	13	(a) Report numbers of individuals at each stage of study—e.g. numbers potentially eligible, examined for eligibility, confirmed eligible, included in the study, completing follow-up, and analyzed	Page 9, lines 168-172
		(b) Give reasons for non-participation at each stage	Page 9, lines 168-172
		(c) Consider use of a flow diagram	Page 9, lines 168-172
**Descriptive data**	14	(a) Give characteristics of study participants (e.g. demographic, clinical, social) and information on exposures and potential confounders	Page 9, General characteristics
		(b) Indicate number of participants with missing data for each variable of interest	N/A

		(c) *Cohort study*—Summarize follow-up time (e.g., average and total amount)	Page 9, lines 177-179
**Outcome data**	15	*Cohort study*—Report numbers of outcome events or summary measures over time	Page 9, lines 179-180<br><br>Page 11, Survival analysis
		Case-control study—Report numbers in each exposure category, or summary measures of exposure	N/A
		Cross-sectional study—Report numbers of outcome events or summary measures	N/A
**Main results**	16	(*a*) Give unadjusted estimates and, if applicable, confounder-adjusted estimates and their precision (e.g., 95% confidence interval). Make clear which confounders were adjusted for and why they were included	Pages 11-13, Factors associated with ICU mortality
		(*b*) Report category boundaries when continuous variables were categorized	Pages 9-10, Table 1
		(*c*) If relevant, consider translating estimates of relative risk into absolute risk for a meaningful time period	N/A
**Other analyses**	17	Report other analyses done—e.g. analyses of subgroups and interactions, and sensitivity analyses	Pages 13-14, lines 230-240 (S5-S6 Tables)
**Discussion**			
**Key results**	18	Summarize key results with reference to study objectives	Page 14, lines 243-249
**Limitations**	19	Discuss limitations of the study, taking into account sources of potential bias or imprecision. Discuss both direction and magnitude of any potential bias	Page 17, lines 313-322
**Interpretation**	20	Give a cautious overall interpretation of results considering objectives, limitations, multiplicity of analyses, results from similar studies, and other relevant evidence	Pages 14-17, lines 250-307
**Generalizability**	21	Discuss the generalizability (external validity) of the study results	Page 17, lines 320-322
**Other information**			
**Funding**	22	Give the source of funding and the role of the funders for the present study and, if applicable, for the original study on which the present article is based	N/A

S2 Appendix. Assessment of coma and impaired consciousness.

The Glasgow Coma Scale (GCS) was used at ICU admission to assess the level of consciousness in patients with acute medical, surgical, or traumatic conditions. The GCS evaluates patient responsiveness across three components: best eye response (E), best verbal response (V), and best motor response (M). Each component is scored from 1 (no response) to its maximum normal value: 4 for eye response, 5 for verbal response, and 6 for motor response.

- **Eye response (E)**: 1 = no eye opening; 2 = eye opening in response to pain; 3 = eye opening to sound or command; 4 = spontaneous eye opening.
- **Verbal response (V)**: 1 = no verbal response; 2 = incomprehensible sounds; 3 = inappropriate words; 4 = confused responses; 5 = oriented responses.
- **Motor response (M)**: 1 = no movement; 2 = abnormal extension to pain; 3 = abnormal flexion to pain; 4 = withdrawal from pain; 5 = localization of pain; 6 = obeys commands.

The total GCS score is the sum of the three components and ranges from 3 (deep coma) to 15 (normal level of consciousness). Based on total score, impairment can be classified as mild (GCS score 13–14), moderate (GCS score 9–12), or severe/coma (GCS score 3–8).

S3 Appendix. Variable dictionary.

Variable names	Descriptions	Data types	Coding / Units	Categorization
Outcome				
**death**	Death occurring during ICU stay	Categorical	No / Yes	
Independent variables				
	**Sociodemographic characteristics**			
**age**	Age of the patient at ICU admission	Numeric	years	1 = 18–59 years; 2 = ≥60 years
**sex**	Sex of the patient	Categorical	F = Female; M = Male	
	**Comorbidity- and lifestyle-related factors**			
**hypertension**	History of arterial hypertension prior to ICU admission	Categorical	No / Yes	
**diabetes**	History of diabetes mellitus prior to ICU admission	Categorical	No / Yes	
**TBI**	History of traumatic brain injury (TBI) prior to ICU admission	Categorical	No / Yes	

**prior_surgery**	History of surgery prior to ICU admission	Categorical	No / Yes	
**alcohol**	Alcohol consumption prior to ICU admission	Categorical	No / Yes	
**tobacco**	Tobacco use prior to ICU admission	Categorical	No / Yes	
	**Clinical characteristics at admission**			
**admission_delay**	Time from onset of impaired consciousness to ICU admission	Numeric	days (rounded down to the nearest day; a value of 0 indicates same-day admission, i.e., onset and ICU admission occurred within the same calendar day)	1 = <1 day;<br>2 = ≥1 day
**temperature**	Axillary body temperature at ICU admission	Numeric	°C	0 = Normal temperature (35.1–37.9 °C);<br>1 = Hypothermia (≤35 °C);<br>2 = Hyperthermia (≥38 °C)
**systolic_BP**	Systolic blood pressure measured at ICU admission	Numeric	mmHg	0 = Normal (91–139 mmHg);<br>1 = Hypotension (≤90 mmHg);<br>2 = Hypertension (≥140 mmHg)
**diastolic_BP**	Diastolic blood pressure measured at ICU admission	Numeric	mmHg	0 = Normal (51–89 mmHg);<br>1 = Hypotension (≤50 mmHg);<br>2 = Hypertension (≥90 mmHg)
**saturation**	Peripheral oxygen saturation at ICU admission	Numeric	%	0 = Normal (≥95%);<br>1 = Desaturation (<95%)
**GCS_score**	Glasgow Coma Scale score at ICU admission	Numeric	3–15 (theoretical scale range)	1 = Mild impairment (GCS score 14–13);<br>2 = Moderate impairment (GCS score 12–9);<br>3 = Severe impairment/Coma (GCS score 8–3)

**cause_impaired_consciousness**	Primary cause of impaired consciousness based on clinical assessment and patient records	Categorical	1 = Tumor-related;<br>2 = Vascular;<br>3 = Infectious;<br>4 = Toxic/Drug-related;<br>5 = Metabolic;<br>6 = Endocrine (no cases recorded in this dataset);<br>7 = Traumatic;<br>8 = Seizure-related;<br>9 = Other (anoxic brain injury, electrical injury, etc.);<br>10 = Undetermined	1 = Non-traumatic;<br>2 = Traumatic
**ICU_stay**	Total duration of ICU stay	Numeric	days	
	**Management-related variables**			
**intubation**	Orotracheal intubation performed during ICU stay	Categorical	No / Yes	
**oxygen_therapy**	Oxygen therapy administered during ICU stay	Categorical	No / Yes	
**mechanical_ventilation**	Mechanical ventilation performed during ICU stay	Categorical	No / Yes	
**dialysis**	Renal replacement therapy performed during ICU stay	Categorical	No / Yes	
**sedation**	Administration of sedative medications during ICU stay (e.g., fentanyl, diazepam, midazolam, thiopental)	Categorical	No / Yes	
**blood_transfusion**	Transfusion of labile blood products during ICU stay (e.g., packed red blood cells, fresh frozen plasma,	Categorical	No / Yes	

	platelet concentrates, or whole blood)			

S4 Table. Comparison of samples.

		Initial sample	Final sample	p-value
**N**		**427**	**416**	
Age, median (IQR)		45.0 (30.0–60.0)	45.0 (30.0–60.0)	0.87
Sex, n (%)	Female	219 (51.3)	214 (51.4)	> 0.99
	Male	208 (48.7)	202 (48.6)	
Hypertension, n (%)	Yes	169 (39.6)	165 (39.7)	> 0.99
	No	258 (60.4)	251 (60.3)	
Diabetes, n (%)	Yes	59 (13.8)	59 (14.2)	0.96
	No	368 (86.2)	357 (85.8)	
TBI, n (%)	Yes	86 (20.1)	84 (20.2)	> 0.99
	No	341 (79.9)	332 (79.8)	
Prior surgery, n (%)	Yes	110 (25.8)	108 (26.0)	> 0.99
	No	317 (74.2)	308 (74.0)	
Alcohol consumption, n (%)	Yes	148 (34.7)	146 (35.1)	0.95
	No	279 (65.3)	270 (64.9)	
Tobacco use, n (%)	Yes	14 (3.3)	12 (2.9)	0.89
	No	413 (96.7)	404 (97.1)	
Admission delay, median (IQR)		1.0 (0–3.0)	1.0 (0–3.0)	0.98
Body temperature, median (IQR)		37.6 (37.0–38.5)	37.6 (37.0–38.5)	0.97
Systolic BP, median (IQR)		135.0 (111.0–164.0)	135.5 (111.8–164.2)	0.83
Diastolic BP, median (IQR)		82.0 (64.0–100.0)	82.0 (64.7–100.0)	0.84
Oxygen saturation, median (IQR)		99.0 (96.0–100)	99.0 (96.7–100)	0.78
Glasgow Coma Scale score, median (IQR)		8.0 (5.0–10.0)	8.0 (6.0–10.0)	0.77
Cause of impaired consciousness, n (%)	Non-traumatic	346 (81.0)	337 (81.0)	> 0.99
	Traumatic	81 (19.0)	79 (19.0)	
ICU length of stay, median (IQR)		3.0 (1.0–7.0)	3.0 (1.0–7.0)	0.62
Orotracheal intubation, n (%)	Yes	306 (71.7)	295 (70.9)	0.87
	No	121 (28.3)	121 (29.1)	
Oxygen therapy, n (%)	Yes	253 (59.3)	248 (59.6)	0.97
	No	174 (40.7)	168 (40.4)	
Mechanical ventilation, n (%)	Yes	261 (61.1)	253 (60.8)	0.98
	No	166 (38.9)	163 (39.2)	
Dialysis, n (%)	Yes	5 (1.2)	5 (1.2)	> 0.99
	No	422 (98.8)	411 (98.8)	
Sedation, n (%)	Yes	138 (32.3)	137 (32.9)	0.91
	No	289 (67.7)	279 (67.1)	
Blood transfusion, n (%)	Yes	55 (12.9)	54 (13.0)	> 0.99
	No	372 (87.1)	362 (87.0)	
Death, n (%)	Yes	290 (67.9)	279 (67.1)	0.85
	No	137 (32.1)	137 (32.9)	

n (%), frequency (percentage); median (IQR), median (interquartile range); TBI, traumatic brain injury; Systolic BP, systolic blood pressure; Diastolic BP, diastolic blood pressure.

S5 Table. Sensitivity analysis excluding patients with traumatic brain injury.

		Standard			Effect type
		aHR	95% CI	p-value	
Time-varying effects					
Age (years)		-	-	0.072	Time-varying effect
DBP (mmHg)		-	-	0.250	Time-varying effect
**Glasgow Coma Scale score**		-	-	**0.006**[a]	Time-varying effect
Fixed effects					
Hypertension	No	1*			
	Yes	0.89	[0.65–1.21]	0.458	Fixed effect
Admission delay (days)		1.01	[0.97–1.05]	0.587	Fixed effect
Body temperature (°C)		1.09	[0.97–1.22]	0.164	Fixed effect
**SBP** (mmHg)		**0.99**	**[0.99–1.00]**	**< 0.001**[a]	Fixed effect
Oxygen saturation (%)		0.97	[0.95–1.00]	0.030	Fixed effect
**Oxygen therapy**	No	1*			
	**Yes**	**0.60**	**[0.44–0.82]**	**0.001**[a]	Fixed effect
Blood transfusion	No	1*			
	Yes	0.65	[0.43–0.97]	0.036	Fixed effect
Stratified variables					
Orotracheal intubation		-	-	-	Stratification
Mechanical ventilation		-	-	-	Stratification

** Reference category; DBP, diastolic blood pressure; SBP, systolic blood pressure; [a] Significant at the 0.05 level.*

S6 Table. Subgroup analyses stratified by Glasgow Coma Scale score (≤ 8 vs > 8).

		GCS score ≤ 8		GCS score > 8		
**event / N**		**201 / 246**		**78 / 170**		
		aHR	95% CI	aHR	95% CI	Effect type
Time-varying effects						
Age (years)		-	-	-	-	Time-varying effect
DBP (mmHg)		-	-	-	-	Time-varying effect
Fixed effects						
Hypertension	No	1*		1*		
	Yes	0.99	[0.70–1.39]	0.74	[0.42–1.31]	Fixed effect
TBI	No	1*		1*		
	Yes	0.72	[0.49–1.06]	0.55	[0.27–1.14]	Fixed effect
Admission delay (days)		1.00	[0.96–1.05]	0.97	[0.90–1.04]	Fixed effect
Body temperature (°C)		1.11	[0.99–1.25]	1.21	[0.94–1.56]	Fixed effect
**SBP** (mmHg)		**0.99**	**[0.99–1.00]**	0.99	[0.98–1.00]	Fixed effect
Oxygen saturation (%)		0.98	[0.96–1.01]	0.97	[0.91–1.04]	Fixed effect
**Oxygen therapy**	No	1*		1*		
	**Yes**	**0.55**	**[0.38–0.78]**	0.76	[0.40–1.44]	Fixed effect
Blood transfusion	No	1*		1*		
	Yes	0.66	[0.39–1.10]	0.89	[0.46–1.73]	Fixed effect
Stratified variables						
Orotracheal intubation		-	-	-	-	Stratification
Mechanical ventilation		-	-	-	-	Stratification

** Reference category; DBP, diastolic blood pressure; TBI, traumatic brain injury; SBP, systolic blood pressure.*
