# Supplementary material for: Survival and factors associated with intensive care unit mortality among adults with impaired consciousness in Benin, a low-resource setting: A retrospective cohort study
Source: PLoS One. 2026 Jun 24;21(6):e0350120. doi: 10.1371/journal.pone.0350120 (PMC13293454; doi:10.1371/journal.pone.0350120)
Supplement: Attachment — Submitted filename: Response_to_Reviewers.docx [file pone.0350120.s003.docx]

# **Point-by-point responses to reviewers’ comments**

Dear Academic Editor and Reviewers,

We thank you for your careful review of our manuscript and for your constructive comments. We have revised the manuscript accordingly and addressed all points raised.

Below, we provide point-by-point responses to each comment. Reviewer comments are presented in italics, followed by our responses in dark blue.

A revised version of the manuscript with tracked changes has been provided to facilitate review, along with a clean version.

We hope that the revised manuscript is now suitable for publication in PLOS ONE.

Sincerely,

**Mahunan Gerard Sossou**

---------------------------------------------------------------------------------------------------------------

## **Responses to Journal Requirements**

*Comment 0: Instruction to include any updated Financial Disclosure statement in the cover letter if modifications are made.*

Response: We thank the editorial office for this instruction. The Financial Disclosure statement has been reviewed, corrected where necessary, and updated accordingly. The revised statement has been included in the cover letter as requested.

*Comment 1: PLOS ONE style requirements, including file naming.*

Response: We have revised the manuscript to comply with the journal’s style requirements, including file naming.

*Comment 2: Inconsistency in the ‘Funding Information’ and ‘Financial Disclosure’ sections regarding grant numbers.*

Response: We have clarified the funding statements to address the inconsistency. This study was conducted using personal resources in Benin. The ARES scholarship supported only the completion of the CU-SDSM training and the preparation of the manuscript; it did not fund the study. Accordingly, we updated the Funding Information and Financial Disclosure sections to indicate that no specific funding was received for this study.

*Comment 3: Issues regarding data sharing restrictions and clarification of the Data Availability Statement in line with journal policy.*

Response: We have addressed the journal’s data sharing requirements by depositing the anonymized dataset on Zenodo (<https://doi.org/10.5281/zenodo.19342169>), ensuring reproducibility of the analyses. The Data Availability Statement has been updated accordingly.

*Comment 4: Clarification on whether interview transcripts were used and on consent for publication of participant data.*

Response: We confirm that this study did not involve interview transcripts. The analysis was conducted using an existing fully anonymized ICU database accessed in February 2025. As no qualitative interviews or identifiable participant data were used, issues related to consent for publication of interview transcripts are not applicable.

*Comment 5: Recommendation to evaluate suggested references for relevance and citation.*

Response: We carefully evaluated the recommended references and incorporated the relevant one into the revised manuscript. In addition, we added further relevant citations to strengthen and align the manuscript with the reviewer’s recommendations.

*Comment 6: Reminder to ensure compliance of all figures with journal technical requirements and recommended use of PLOS figure preparation tools (NAAS) to verify publication quality standards.*

Response: We thank the editorial office for this reminder. All figures have been carefully reviewed and revised as necessary to ensure full compliance with the journal’s technical requirements. We have also used the NAAS figure assessment tool to verify that all figures meet publication-quality standards in accordance with the journal guidelines.

---------------------------------------------------------------------------------------------------------------

## **Responses to Reviewer 1**

*Comment 0: Overall positive assessment of study relevance and methodological approach, highlighting its contribution to underreported critical care outcomes in low-resource settings.*

Response: We thank the reviewer for this positive assessment and for recognizing the relevance of our study. We appreciate the acknowledgment of its contribution to the limited evidence on critical care outcomes in low-resource settings.

*Comment 1: Recognition of study strengths, including relevance of the setting, adequate sample size, appropriate statistical methods, and adherence to reporting standards.*

Response: We thank the reviewer for highlighting the strengths of our study. We appreciate the recognition of the study design, statistical approach, and adherence to STROBE reporting guidelines.

*Comment 2: Identification of major methodological and interpretative limitations, including confounding, potential selection bias, incomplete characterization of ICU context, and issues in the interpretation of management-related variables and survival analysis reporting.*

Response: We thank the reviewer for these important and constructive comments. We acknowledge the methodological and interpretative limitations highlighted, and we have revised the manuscript to clarify these aspects, particularly regarding confounding, interpretation of management-related variables, and reporting of survival analyses.

*Comment 3: Issues related to abstract clarity, including missing study design details, inconsistent terminology, and potential overinterpretation of conclusions regarding ICU resource limitations.*

Response: We thank the reviewer for these helpful comments. The abstract has been revised to specify a single-center retrospective cohort design. “Coma” has been replaced with “impaired consciousness” to better reflect the study population, which included patients with mild, moderate, and severe impairment of consciousness. The concluding statement has been revised to reduce overinterpretation and align it with the observational nature of the study.

*Comment 4: Insufficient literature review and unclear conceptual framing of the study focus between coma epidemiology and ICU capacity in low-resource settings.*

Response: The Introduction has been revised to clarify the conceptual framework, focusing on outcomes of patients with impaired consciousness in a resource-limited ICU setting. The literature review has been expanded to include the reference suggested by the reviewer (Baker et al.), as well as other relevant studies on ICU outcomes in low- and middle-income countries.

*Comment 5: Methodological clarifications regarding the study database timeline and construction, inclusion criteria, key prognostic variables, and confounding by indication in the statistical interpretation.*

Response: We thank the reviewer for these important comments.

The Methods section has been revised to clarify the study database timeline and construction (page 6, lines 114–120). Data were accessed in February 2025 from an anonymized ICU database that had been constructed in 2019 using archived medical records of patients who were discharged from, or died in, the ICU between 2015 and 2017. The database was developed by a trained team using a pretested questionnaire, and the dataset was anonymized before access in 2025.

The inclusion criteria have also been clarified (page 5, lines 87–93). Patients with a Glasgow Coma Scale (GCS) score of 3–14 were included to capture the full spectrum of impaired consciousness in an ICU setting admitting patients with varying levels of impaired consciousness, rather than restricting the analysis to coma alone.

In addition, we acknowledge that several key prognostic variables highlighted by the reviewer were not available in the dataset, and this limitation has been explicitly addressed in the Discussion section (page 17, lines 313–314).

Finally, the manuscript has been revised to avoid causal interpretation of management-related variables and to account for confounding by indication, particularly regarding oxygen therapy (page 16, lines 282–286).

*Comment 6: Potential misinterpretation of the association between oxygen therapy and ICU mortality, with concern for confounding by indication.*

Response: We thank the reviewer for this important comment. The manuscript has been revised to avoid causal interpretation of the association between oxygen therapy and ICU mortality. The findings are now presented as an association rather than a protective effect, acknowledging the potential influence of confounding by indication. This clarification has been incorporated in the Discussion section (page 16, lines 282–286; page 17, lines 314–316).

*Comment 7: Insufficient contextual interpretation of the short median survival time, including possible contributions of late ICU admission, limited life-support resources, severe brain injury, and absence of neurocritical care.*

Response: We thank the reviewer for this helpful comment. The interpretation of the short median survival time has been revised in the Discussion to better reflect possible contributing factors, including delayed ICU admission, limited life-support resources, and the absence of neurocritical care. This clarification is provided in the revised manuscript (page 15, lines 261–267).

*Comment 8: Limited contextualization of ICU mortality in the Discussion with existing literature, incomplete interpretation of the TBI association, and overstatement of the implications of oxygen therapy for survival.*

Response: We thank the reviewer for this thoughtful comment.

The Discussion has been revised to better contextualize ICU mortality by adding a Nigerian reference and broader comparisons with sub-Saharan African ICU mortality, typically reported in the 30–50% range. We also discuss possible contributing factors related to health system capacity and delayed ICU admission (page 15, lines 262–267).

Regarding traumatic brain injury (TBI), the manuscript already included explanations consistent with those raised by the reviewer (including selection bias and patient-related factors). These points have been further clarified and reinforced in the revised Discussion (page 16, lines 289–292) to improve clarity and alignment with the reviewer’s interpretation.

Finally, the language regarding oxygen therapy has been softened to remain consistent with the observational nature of the study (page 16, lines 282–286).

*Comment 9: Incomplete reporting of key study limitations, including confounding by indication, selection bias, lack of cause-of-coma classification, and absence of validated severity scores (e.g., APACHE/SOFA).*

Response: We thank the reviewer for this important comment.

The Limitations section has been revised to explicitly acknowledge the absence of validated severity scores (e.g., APACHE/SOFA), which were not available in the dataset, and the potential confounding by indication affecting interpretation of management-related variables. We also clarify that inclusion of ICU patients only may limit the generalizability of the findings (page 17, lines 313–322).

In addition, the classification of the cause of impaired consciousness was clinically assessed but not reported in the original manuscript. This has now been added to Table 1 in the revised version (page 10).

*Comment 10: Overstatement in the conclusion regarding the effect of oxygen therapy on mortality, requiring revision to avoid causal implication and align with observational evidence.*

Response: We thank the reviewer for this important comment. The Conclusion has been revised to avoid overstatement regarding the effect of oxygen therapy on mortality. The wording has been softened to reflect the observational nature of the study and to avoid causal interpretation, in line with the available evidence. The revised conclusion now emphasizes oxygen availability as a priority area for strengthening essential critical care rather than a direct mortality-reducing intervention.

*Comment 11: Minor issue regarding interpretability of continuous variables in Table 2 due to reporting of hazard ratios per unit increase, with recommendation for rescaling (e.g., SBP per 10 mmHg).*

Response: We thank the reviewer for this helpful comment. To ensure consistency across all analyses, continuous variables, including SBP and DBP, were retained in their original units in Table 2 (univariable Cox model). In the multivariable model (Table 3), SBP was the only blood pressure variable that remained independently and significantly associated with the outcome. For improved clinical interpretability, its effect was additionally rescaled in the Discussion to reflect a 10-mmHg increase by multiplying the regression coefficient (log-HR) by 10 before exponentiation, with the corresponding 95% CI recalculated on the log scale (page 15, lines 274–275). This rescaling was applied solely for interpretative purposes and did not modify the underlying statistical models.

*Comment 12: Key revisions needed including clarification of cohort definition (GCS score 9–14), strengthening of the literature review with African ICU studies, cautious interpretation of management-related variables due to confounding by indication, expansion of study limitations, clarification of database creation, and improved contextualization of high ICU mortality.*

Response: We thank the reviewer for this synthesis of key required revisions. The manuscript has been substantially revised accordingly. Cohort definition has been clarified, including justification of the inclusion of patients with GCS score 9–14. The literature review has been strengthened with additional African ICU studies to improve contextualization. Management-related variables are now interpreted cautiously, with explicit consideration of confounding by indication. The limitations section has been expanded to reflect key methodological constraints. The study database creation process has been clarified to improve transparency. Finally, the Discussion has been revised to better contextualize the high ICU mortality in relation to existing literature and limited ICU capacity.

---------------------------------------------------------------------------------------------------------------

## **Responses to Reviewer 2**

*Comment 1: Misalignment between the Introduction and the study focus, with excessive emphasis on critical care systems rather than on impaired consciousness as a clinical entity and its epidemiology.*

Response: We thank the reviewer for this helpful comment. The Introduction has been revised to place greater emphasis on impaired consciousness as a clinical entity and on its epidemiology, in line with the title and objectives of the manuscript. The broader discussion of critical care systems has been streamlined and retained only where needed for context in resource-limited settings.

*Comment 2: Need for clarification of the use of data from 2015–2017 and justification of the current relevance of the findings given potential changes in ICU capacity and practices over time.*

Response: We thank the reviewer for this important comment. The use of data from 2015–2017 reflects the availability of an ICU database constructed from archived medical records, as this ICU does not routinely maintain a structured research registry. These findings should therefore be interpreted in the context of the limited critical care capacity typical of many low- and middle-income countries during the study period. Although the data are based on ICU admissions from 2015 to 2017, they remain an important source of information on ICU outcomes among adults with impaired consciousness in Benin. This study therefore contributes valuable evidence in a still under-described clinical setting, and this temporal limitation has been acknowledged in the revised manuscript.

*Comment 3: Need for clarification of the definition of impaired consciousness, given the inclusion of a wide GCS range (3–14), introducing clinical heterogeneity, and recommendation to discuss this limitation and consider sensitivity analyses.*

Response: We thank the reviewer for this thoughtful comment.

The study included all patients with a Glasgow Coma Scale (GCS) score of 3–14 at ICU admission to capture the full spectrum of impaired consciousness encountered in routine practice at this ICU. The different levels of impaired consciousness (mild, moderate and severe) are now clearly taken into account in Table 1 (page 10).

To address the concern about clinical heterogeneity, we performed subgroup analyses stratified by GCS score (≤ 8 vs > 8). However, only the subgroup with GCS score ≤ 8 had a sufficient number of events to allow reliable subgroup interpretation, whereas the higher GCS subgroup was underpowered for stable estimates (page 14, lines 235–240).

*Comment 4: Need to emphasize the short median survival time as a marker of high early mortality and better link this finding to potential gaps in early critical care delivery.*

Response: We thank the reviewer for this important comment. The short median time to death in the ICU (5 days) has been further emphasized as reflecting early ICU mortality. This interpretation has been strengthened by more explicitly linking this pattern to limited ICU resources, the absence of neurocritical care, and delayed access to intensive care in this resource-limited setting (page 15, lines 261–264).

*Comment 5: Need to revise the opening of the Discussion to restate the study aim and provide a clearer conceptual summary of the main findings.*

Response: We thank the reviewer for this helpful comment. The opening section of the Discussion has been revised to restate the aim of the study and to provide a clearer conceptual summary of the main findings, thereby improving the alignment between the study objectives and the interpretation of the results (page 14, lines 243–249).

*Comment 6: Inconsistent use of terminology across the manuscript, including interchangeable use of “impaired consciousness,” “coma,” and “comatose,” as well as lack of clear distinction between “ICU mortality” and “in-hospital mortality,” and between outcome-related time metrics (survival time, time to death, ICU length of stay). Need for standardization of key terms throughout the manuscript.*

Response: We thank the reviewer for this important comment. The manuscript has been carefully revised to standardize terminology throughout. “Impaired consciousness” is now consistently used to describe the study population, in line with the inclusion criteria, while “coma” and “comatose” have been removed or restricted to appropriate clinical contexts. We have also standardized outcome definitions, ensuring consistent use of “ICU mortality” and removing “in-hospital mortality” to avoid ambiguity. Similarly, time-related outcomes have been harmonized, with clear and consistent use of “time to death in the ICU,” and “ICU length of stay” is now used consistently to describe duration of ICU stay. These revisions improve clarity and internal consistency across all sections of the manuscript.

---------------------------------------------------------------------------------------------------------------

## **Responses to Reviewer 3**

*Comment 1: Overall positive assessment of the manuscript, with no major concerns and recognition of its value in a low-resource setting.*

Response: We thank the reviewer for this positive evaluation of our manuscript and for recognizing the relevance of our findings in a low-resource setting. We are grateful for the constructive assessment and have carefully addressed all comments raised in the review process.
