# Supplementary material for: Survival and factors associated with intensive care unit mortality among adults with impaired consciousness in Benin, a low-resource setting: A retrospective cohort study
Source: PLoS One. 2026 Jun 24;21(6):e0350120. doi: 10.1371/journal.pone.0350120 (PMC13293454; doi:10.1371/journal.pone.0350120)
Supplement: Peer review history

# Peer review: Decision Letter 0

**DOI:** 10.1371/journal.pone.0350120.r001

**Authors:** Nik Hisamuddin Nik Ab. Rahman

25 Mar 2026

PONE-D-26-08886

In-hospital survival and factors associated with mortality among adults with impaired consciousness in intensive care in Benin, a low-resource setting: a retrospective cohort study

PLOS One

Dear Dr. Sossou,

Thank you for submitting your manuscript to PLOS ONE. After careful consideration, we feel that it has merit but does not fully meet PLOS ONE’s publication criteria as it currently stands. Therefore, we invite you to submit a revised version of the manuscript that addresses the points raised during the review process.

Please submit your revised manuscript by May 09 2026 11:59PM. If you will need more time than this to complete your revisions, please reply to this message or contact the journal office at plosone@plos.org. When you're ready to submit your revision, log on to https://www.editorialmanager.com/pone/ and select the 'Submissions Needing Revision' folder to locate your manuscript file.

Please include the following items when submitting your revised manuscript:

- A letter that responds to each point raised by the academic editor and reviewer(s). You should upload this letter as a separate file labeled 'Response to Reviewers'.
- A marked-up copy of your manuscript that highlights changes made to the original version. You should upload this as a separate file labeled 'Revised Manuscript with Track Changes'.
- An unmarked version of your revised paper without tracked changes. You should upload this as a separate file labeled 'Manuscript'.

If you would like to make changes to your financial disclosure, please include your updated statement in your cover letter. Guidelines for resubmitting your figure files are available below the reviewer comments at the end of this letter.

If applicable, we recommend that you deposit your laboratory protocols in protocols.io to enhance the reproducibility of your results. Protocols.io assigns your protocol its own identifier (DOI) so that it can be cited independently in the future. For instructions see: https://journals.plos.org/plosone/s/submission-guidelines#loc-laboratory-protocols. Additionally, PLOS ONE offers an option for publishing peer-reviewed Lab Protocol articles, which describe protocols hosted on protocols.io. Read more information on sharing protocols at https://plos.org/protocols?utm_medium=editorial-email&utm_source=authorletters&utm_campaign=protocols.

As the corresponding author, your ORCID iD is verified in the submission system and will appear in the published article. PLOS supports the use of ORCID, and we encourage all coauthors to register for an ORCID iD and use it as well. Please encourage your coauthors to verify their ORCID iD within the submission system before final acceptance, as unverified ORCID iDs will not appear in the published article. *Only*   the individual author can complete the verification step; PLOS staff *cannot*   verify ORCID iDs on behalf of authors.

We look forward to receiving your revised manuscript.

Kind regards,

Nik Hisamuddin Nik Ab. Rahman

Academic Editor

PLOS One

**Journal Requirements:**

1. When submitting your revision, we need you to address these additional requirements.

Please ensure that your manuscript meets PLOS ONE's style requirements, including those for file naming. The PLOS ONE style templates can be found at

https://journals.plos.org/plosone/s/file?id=wjVg/PLOSOne_formatting_sample_main_body.pdf and

https://journals.plos.org/plosone/s/file?id=ba62/PLOSOne_formatting_sample_title_authors_affiliations.pdf

2. We note that the grant information you provided in the ‘Funding Information’ and ‘Financial Disclosure’ sections do not match.

When you resubmit, please ensure that you provide the correct grant numbers for the awards you received for your study in the ‘Funding Information’ section.

3. We note that you have indicated that there are restrictions to data sharing for this study. For studies involving human research participant data or other sensitive data, we encourage authors to share de-identified or anonymized data. However, when data cannot be publicly shared for ethical reasons, we allow authors to make their data sets available upon request. For information on unacceptable data access restrictions, please see http://journals.plos.org/plosone/s/data-availability#loc-unacceptable-data-access-restrictions.

Before we proceed with your manuscript, please address the following prompts:

a) If there are ethical or legal restrictions on sharing a de-identified data set, please explain them in detail (e.g., data contain potentially identifying or sensitive patient information, data are owned by a third-party organization, etc.) and who has imposed them (e.g., a Research Ethics Committee or Institutional Review Board, etc.). Please also provide contact information for a data access committee, ethics committee, or other institutional body to which data requests may be sent.

b) If there are no restrictions, please upload the minimal anonymized data set necessary to replicate your study findings to a stable, public repository and provide us with the relevant URLs, DOIs, or accession numbers. Please see http://www.bmj.com/content/340/bmj.c181.long for guidelines on how to de-identify and prepare clinical data for publication. For a list of recommended repositories, please see https://journals.plos.org/plosone/s/recommended-repositories. You also have the option of uploading the data as Supporting Information files, but we would recommend depositing data directly to a data repository if possible.

Please update your Data Availability statement in the submission form accordingly.

4. We note that this data set consists of interview transcripts. Can you please confirm that all participants gave consent for interview transcript to be published?

If they DID provide consent for these transcripts to be published, please also confirm that the transcripts do not contain any potentially identifying information (or let us know if the participants consented to having their personal details published and made publicly available). We consider the following details to be identifying information:

- Names, nicknames, and initials

- Age more specific than round numbers

- GPS coordinates, physical addresses, IP addresses, email addresses

- Information in small sample sizes (e.g. 40 students from X class in X year at X university)

- Specific dates (e.g. visit dates, interview dates)

- ID numbers

Or, if the participants DID NOT provide consent for these transcripts to be published:

- Provide a de-identified version of the data or excerpts of interview responses

- Provide information regarding how these transcripts can be accessed by researchers who meet the criteria for access to confidential data, including:

a) the grounds for restriction

b) the name of the ethics committee, Institutional Review Board, or third-party organization that is imposing sharing restrictions on the data

c) a non-author, institutional point of contact that is able to field data access queries, in the interest of maintaining long-term data accessibility.

d) Any relevant data set names, URLs, DOIs, etc. that an independent researcher would need in order to request your minimal data set.

For further information on sharing data that contains sensitive participant information, please see: https://journals.plos.org/plosone/s/data-availability#loc-human-research-participant-data-and-other-sensitive-data

If there are ethical, legal, or third-party restrictions upon your dataset, you must provide all of the following details (https://journals.plos.org/plosone/s/data-availability#loc-acceptable-data-access-restrictions):

a) A complete description of the dataset

b) The nature of the restrictions upon the data (ethical, legal, or owned by a third party) and the reasoning behind them

c) The full name of the body imposing the restrictions upon your dataset (ethics committee, institution, data access committee, etc)

d) If the data are owned by a third party, confirmation of whether the authors received any special privileges in accessing the data that other researchers would not have

e) Direct, non-author contact information (preferably email) for the body imposing the restrictions upon the data, to which data access requests can be sent

5. If the reviewer comments include a recommendation to cite specific previously published works, please review and evaluate these publications to determine whether they are relevant and should be cited. There is no requirement to cite these works unless the editor has indicated otherwise.

[Note: HTML markup is below. Please do not edit.]

Reviewers' comments:

Reviewer's Responses to Questions

**Comments to the Author**

1. Is the manuscript technically sound, and do the data support the conclusions?

The manuscript must describe a technically sound piece of scientific research with data that supports the conclusions. Experiments must have been conducted rigorously, with appropriate controls, replication, and sample sizes. The conclusions must be drawn appropriately based on the data presented.

Reviewer #1: Partly

Reviewer #2: Yes

Reviewer #3: Yes

**********

2. Has the statistical analysis been performed appropriately and rigorously?

Reviewer #1: No

Reviewer #2: Yes

Reviewer #3: Yes

**********

3. Have the authors made all data underlying the findings in their manuscript fully available?

The PLOS Data policy requires authors to make all data underlying the findings described in their manuscript fully available without restriction, with rare exception (please refer to the Data Availability Statement in the manuscript PDF file). The data should be provided as part of the manuscript or its supporting information, or deposited to a public repository. For example, in addition to summary statistics, the data points behind means, medians and variance measures should be available. If there are restrictions on publicly sharing data—e.g. participant privacy or use of data from a third party—those must be specified.

Reviewer #1: Yes

Reviewer #2: No

Reviewer #3: Yes

**********

4. Is the manuscript presented in an intelligible fashion and written in standard English?

PLOS ONE does not copyedit accepted manuscripts, so the language in submitted articles must be clear, correct, and unambiguous. Any typographical or grammatical errors should be corrected at revision, so please note any specific errors here.

Reviewer #1: Yes

Reviewer #2: Yes

Reviewer #3: Yes

**********

5. Review Comments to the Author

Please use the space provided to explain your answers to the questions above. You may also include additional comments for the author, including concerns about dual publication, research ethics, or publication ethics. (Please upload your review as an attachment if it exceeds 20,000 characters)

**Reviewer #1:**  This manuscript presents a retrospective cohort study of ICU patients with impaired consciousness in Benin, evaluating survival and predictors of in-hospital mortality using Kaplan–Meier and Cox proportional hazards models.

The study addresses an important and underreported area: critical care outcomes in low-resource African settings, which aligns with global health priorities such as those promoted by the World Health Organization and World Federation of Intensive and Critical Care Medicine.

A. Strengths

1. Important data from an understudied region

2. Reasonable sample size (n=416)

3. Appropriate use of survival analysis

4. Time-varying effects modeling

5. Bootstrap validation

6. Generally clear manuscript structure

7. STROBE reporting acknowledged

B. Major weaknesses

However, several methodological and interpretative issues limit the study’s impact:

1. Causal interpretation of management variables (oxygen therapy, intubation)

2. Confounding by indication

3. Poor clarity regarding ICU resources

4. Weak characterization of coma etiology

5. Incomplete reporting of survival modeling

6. Potential selection bias

7. Interpretation occasionally overreaches

C. Abstract

Strengths

The abstract is structured and concise.

Issues

1. Missing design details

Should mention:

single-center

retrospective cohort

2. Inconsistent terminology

Use coma consistently.

3. Overinterpretation

The sentence:

suggesting substantial gaps in essential critical care delivery

This is speculative because ICU capacity was not measured.

Better wording:

suggesting possible limitations in essential critical care resources

D. Introduction

Strengths

Good global context

Logical flow

Clear research question

Issues

1. Literature review insufficient

Several key African ICU outcome studies are missing.

Examples that should be referenced:

African ICU mortality reviews

Critical illness outcomes in LMICs

For example studies by:

Adhikari et al.

Riviello et al.

Baker et al.

Without this context, the literature review feels incomplete.

2. Conceptual framing

The study mixes two themes:

coma epidemiology

ICU capacity in LMICs

But does not clearly define which is primary.

Better framing:

Focus should be

outcomes of coma in a resource-limited ICU

E. Methods

This is the strongest section, but still has several issues.

1. Study design

Well described.

However:

Issue

The database creation timeline is confusing.

Data collected:

2015–2017 (patients)

database built 2019

extracted 2025

Add clarification:

Who entered the data?

Was quality checked?

Was double entry performed?

2. Inclusion criteria

GCS 3–14.

This is unusual.

Standard coma definitions are:

GCS ≤ 8.

You should justify why GCS 9–14 were included.

Otherwise the cohort mixes:

coma

altered consciousness

mild neurologic impairment

This increases heterogeneity.

3. Variables

Selection is reasonable.

However major predictors of ICU mortality are missing:

Missing variables:

mechanical ventilation duration

vasopressor use

Infection/sepsis

stroke vs metabolic coma

APACHE or SOFA score

This limits interpretability.

4. Statistical model

Overall well done.

Strengths:

Kaplan–Meier survival

Cox regression

VIF for multicollinearity

bootstrap validation

sensitivity analysis

This is statistically sound.

Major statistical concern

Confounding by indication.

Variables like:

intubation

ventilation

oxygen therapy

reflect severity of illness, not treatment effects.

Example:

Patients receiving oxygen likely had better physiological reserve, not that oxygen reduced mortality.

Therefore:

Interpretation as protective intervention is incorrect.

The manuscript repeatedly implies causality.

This must be corrected.

F. Results

Strengths

Clear presentation.

Major issue: Oxygen therapy finding

Result:

HR = 0.58

Interpreted as protective.

But likely represents:

triage bias

survivorship bias

resource allocation

Example scenario:

patients dying rapidly may never receive oxygen

Therefore the conclusion

oxygen therapy reduces mortality is not supported.

Better interpretation:

receipt of oxygen therapy was associated with lower mortality, possibly reflecting differences in patient severity or resource allocation.

G. Survival analysis

Median survival = 5 days

This is extremely short.

Interpretation should consider:

Possible explanations:

late ICU admission

limited life-support resources

high prevalence of irreversible brain injury

absence of neurocritical care

These are not discussed sufficiently.

H. Discussion

Generally well written.

However there are several issues.

1. Comparison with literature

Comparisons are limited.

Important to contextualize with African ICU mortality rates, which are typically:

30–50%

67% is very high.

Possible reasons should be discussed:

delayed ICU admission

lack of ventilators

lack of imaging

infectious etiologies

2. Interpretation of TBI result

Finding:

TBI protective (HR 0.66)

Explanation given:

selection bias

This is reasonable.

But other explanations:

TBI patients younger

reversible pathology

ICU admission bias

Better discussion needed.

3. Overinterpretation

The paper repeatedly suggests:

strengthening oxygen therapy will improve survival.

This is not proven by observational data.

The language should be softened.

I. Limitations

This section is good but incomplete.

Missing limitations:

1. Confounding by indication

Major issue for treatment variables.

2. Selection bias

Only ICU patients included.

3. Lack of cause-of-coma classification

Major weakness.

4. Lack of severity scores

No APACHE / SOFA.

J. Conclusion

The conclusion is mostly appropriate.

But this line overreaches:

oxygen therapy could substantially reduce preventable deaths

Better:

ensuring reliable oxygen availability may represent a priority area for strengthening essential critical care.

K. Tables

Tables are clear.

Minor issues:

Table 2

Continuous variables reported with HR per unit.

Interpretability could improve if scaled:

Example:

SBP per 10 mmHg.

L. Key revisions needed:

1. Clarify cohort definition

Explain inclusion of GCS 9–14.

2. Improve literature review

Add African ICU studies.

3. Address confounding by indication

Interpret treatment variables cautiously.

4. Expand limitations

5. Clarify database creation

6. Improve discussion of extremely high mortality

**Reviewer #2:**   PONE-D-26-08886

In-hospital survival and factors associated with mortality among adults with impaired consciousness in intensive care in Benin, a low-resource setting: a retrospective cohort study

In this retrospective cohort study, the authors examined in-hospital survival and factors associated with mortality among adults with impaired consciousness admitted to an ICU in Benin. Among 416 patients, mortality was high (67.1%) with a median survival of five days. Mortality was associated with age, GCS score, systolic blood pressure, body temperature, and oxygen therapy.

Comments

The introduction should be more reflective of the title of the Manuscript, the Authors start with a broad discussion on critical care systems, based on the title the focus should be more about coma/impaired consciousness as a clinical entity and its epidemiology.

The Authors should better clarify why the study relies on data from 2015–2017. It is unclear if there is still relevance regarding these findings, as ICU capacity or practices in Benin might have changed since.

The definition of impaired consciousness requires clarifications: The inclusion of patients with GCS 3–14 introduces substantial clinical heterogeneity, particularly between mild impairment and deep coma, this should be discussed by the Authors, and a sensitivity analysis could be considered.

The short median survival time suggests high early mortality. The authors should emphasize this finding and better link it to potential gaps in early critical care delivery.

The first section of the discussion should be revised to include the aim of the study and a conceptual summary of the results

Several terms appear to be used interchangeably throughout the manuscript (“impaired consciousness,” “coma,” and “comatose” are used to describe the study population, despite inclusion criteria spanning GCS 3–14;“in-hospital mortality” and “ICU mortality,” as well as “survival time,” “time to death,” and “ICU length of stay,” are used without clear distinction). The Authors should standardize terminology and ensure consistent use of key terms across all sections of the manuscript

**Reviewer #3:**  The authors have addressed the previous comments well, and the manuscript has improved significantly.

The study is clear, well organized, and the methods are appropriate. The statistical analysis is adequate, and the results support the conclusions. The discussion is balanced and reflects the limitations of the study.

This work provides useful data from a low-resource setting, which is important and adds value to the literature.

I have no major concerns.

**********

6. PLOS authors have the option to publish the peer review history of their article (what does this mean?). If published, this will include your full peer review and any attached files.

If you choose “no”, your identity will remain anonymous but your review may still be made public.

**Do you want your identity to be public for this peer review?**   For information about this choice, including consent withdrawal, please see our Privacy Policy.

Reviewer #1: No

Reviewer #2: No

Reviewer #3: **Yes:**  Abdullah Abbas Saleh Al-Murad

**********

[NOTE: If reviewer comments were submitted as an attachment file, they will be attached to this email and accessible via the submission site. Please log into your account, locate the manuscript record, and check for the action link "View Attachments". If this link does not appear, there are no attachment files.]

To ensure your figures meet our technical requirements, please review our figure guidelines: https://journals.plos.org/plosone/s/figures

You may also use PLOS’s free figure tool, NAAS, to help you prepare publication quality figures: https://journals.plos.org/plosone/s/figures#loc-tools-for-figure-preparation.

NAAS will assess whether your figures meet our technical requirements by comparing each figure against our figure specifications.

# Author comment: Author response to Decision Letter 1

**DOI:** 10.1371/journal.pone.0350120.r002

2 May 2026

Point-by-point responses to reviewers’ comments

Dear Academic Editor and Reviewers,

We thank you for your careful review of our manuscript and for your constructive comments. We have revised the manuscript accordingly and addressed all points raised.

Below, we provide point-by-point responses to each comment. Reviewer comments are presented in italics, followed by our responses in dark blue.

A revised version of the manuscript with tracked changes has been provided to facilitate review, along with a clean version.

We hope that the revised manuscript is now suitable for publication in PLOS ONE.

Sincerely,

Mahunan Gerard Sossou

---------------------------------------------------------------------------------------------------------------

Responses to Journal Requirements

Comment 0: Instruction to include any updated Financial Disclosure statement in the cover letter if modifications are made.

Response: We thank the editorial office for this instruction. The Financial Disclosure statement has been reviewed, corrected where necessary, and updated accordingly. The revised statement has been included in the cover letter as requested.

Comment 1: PLOS ONE style requirements, including file naming.

Response: We have revised the manuscript to comply with the journal’s style requirements, including file naming.

Comment 2: Inconsistency in the ‘Funding Information’ and ‘Financial Disclosure’ sections regarding grant numbers.

Response: We have clarified the funding statements to address the inconsistency. This study was conducted using personal resources in Benin. The ARES scholarship supported only the completion of the CU-SDSM training and the preparation of the manuscript; it did not fund the study. Accordingly, we updated the Funding Information and Financial Disclosure sections to indicate that no specific funding was received for this study.

Comment 3: Issues regarding data sharing restrictions and clarification of the Data Availability Statement in line with journal policy.

Response: We have addressed the journal’s data sharing requirements by depositing the anonymized dataset on Zenodo (https://doi.org/10.5281/zenodo.19342169), ensuring reproducibility of the analyses. The Data Availability Statement has been updated accordingly.

Comment 4: Clarification on whether interview transcripts were used and on consent for publication of participant data.

Response: We confirm that this study did not involve interview transcripts. The analysis was conducted using an existing fully anonymized ICU database accessed in February 2025. As no qualitative interviews or identifiable participant data were used, issues related to consent for publication of interview transcripts are not applicable.

Comment 5: Recommendation to evaluate suggested references for relevance and citation.

Response: We carefully evaluated the recommended references and incorporated the relevant one into the revised manuscript. In addition, we added further relevant citations to strengthen and align the manuscript with the reviewer’s recommendations.

Comment 6: Reminder to ensure compliance of all figures with journal technical requirements and recommended use of PLOS figure preparation tools (NAAS) to verify publication quality standards.

Response: We thank the editorial office for this reminder. All figures have been carefully reviewed and revised as necessary to ensure full compliance with the journal’s technical requirements. We have also used the NAAS figure assessment tool to verify that all figures meet publication-quality standards in accordance with the journal guidelines.

---------------------------------------------------------------------------------------------------------------

Responses to Reviewer 1

Comment 0: Overall positive assessment of study relevance and methodological approach, highlighting its contribution to underreported critical care outcomes in low-resource settings.

Response: We thank the reviewer for this positive assessment and for recognizing the relevance of our study. We appreciate the acknowledgment of its contribution to the limited evidence on critical care outcomes in low-resource settings.

Comment 1: Recognition of study strengths, including relevance of the setting, adequate sample size, appropriate statistical methods, and adherence to reporting standards.

Response: We thank the reviewer for highlighting the strengths of our study. We appreciate the recognition of the study design, statistical approach, and adherence to STROBE reporting guidelines.

Comment 2: Identification of major methodological and interpretative limitations, including confounding, potential selection bias, incomplete characterization of ICU context, and issues in the interpretation of management-related variables and survival analysis reporting.

Response: We thank the reviewer for these important and constructive comments. We acknowledge the methodological and interpretative limitations highlighted, and we have revised the manuscript to clarify these aspects, particularly regarding confounding, interpretation of management-related variables, and reporting of survival analyses.

Comment 3: Issues related to abstract clarity, including missing study design details, inconsistent terminology, and potential overinterpretation of conclusions regarding ICU resource limitations.

Response: We thank the reviewer for these helpful comments. The abstract has been revised to specify a single-center retrospective cohort design. “Coma” has been replaced with “impaired consciousness” to better reflect the study population, which included patients with mild, moderate, and severe impairment of consciousness. The concluding statement has been revised to reduce overinterpretation and align it with the observational nature of the study.

Comment 4: Insufficient literature review and unclear conceptual framing of the study focus between coma epidemiology and ICU capacity in low-resource settings.

Response: The Introduction has been revised to clarify the conceptual framework, focusing on outcomes of patients with impaired consciousness in a resource-limited ICU setting. The literature review has been expanded to include the reference suggested by the reviewer (Baker et al.), as well as other relevant studies on ICU outcomes in low- and middle-income countries.

Comment 5: Methodological clarifications regarding the study database timeline and construction, inclusion criteria, key prognostic variables, and confounding by indication in the statistical interpretation.

Response: We thank the reviewer for these important comments.

The Methods section has been revised to clarify the study database timeline and construction (page 6, lines 114–120). Data were accessed in February 2025 from an anonymized ICU database that had been constructed in 2019 using archived medical records of patients who were discharged from, or died in, the ICU between 2015 and 2017. The database was developed by a trained team using a pretested questionnaire, and the dataset was anonymized before access in 2025.

The inclusion criteria have also been clarified (page 5, lines 87–93). Patients with a Glasgow Coma Scale (GCS) score of 3–14 were included to capture the full spectrum of impaired consciousness in an ICU setting admitting patients with varying levels of impaired consciousness, rather than restricting the analysis to coma alone.

In addition, we acknowledge that several key prognostic variables highlighted by the reviewer were not available in the dataset, and this limitation has been explicitly addressed in the Discussion section (page 17, lines 313–314).

Finally, the manuscript has been revised to avoid causal interpretation of management-related variables and to account for confounding by indication, particularly regarding oxygen therapy (page 16, lines 282–286).

Comment 6: Potential misinterpretation of the association between oxygen therapy and ICU mortality, with concern for confounding by indication.

Response: We thank the reviewer for this important comment. The manuscript has been revised to avoid causal interpretation of the association between oxygen therapy and ICU mortality. The findings are now presented as an association rather than a protective effect, acknowledging the potential influence of confounding by indication. This clarification has been incorporated in the Discussion section (page 16, lines 282–286; page 17, lines 314–316).

Comment 7: Insufficient contextual interpretation of the short median survival time, including possible contributions of late ICU admission, limited life-support resources, severe brain injury, and absence of neurocritical care.

Response: We thank the reviewer for this helpful comment. The interpretation of the short median survival time has been revised in the Discussion to better reflect possible contributing factors, including delayed ICU admission, limited life-support resources, and the absence of neurocritical care. This clarification is provided in the revised manuscript (page 15, lines 261–267).

Comment 8: Limited contextualization of ICU mortality in the Discussion with existing literature, incomplete interpretation of the TBI association, and overstatement of the implications of oxygen therapy for survival.

Response: We thank the reviewer for this thoughtful comment.

The Discussion has been revised to better contextualize ICU mortality by adding a Nigerian reference and broader comparisons with sub-Saharan African ICU mortality, typically reported in the 30–50% range. We also discuss possible contributing factors related to health system capacity and delayed ICU admission (page 15, lines 262–267).

Regarding traumatic brain injury (TBI), the manuscript already included explanations consistent with those raised by the reviewer (including selection bias and patient-related factors). These points have been further clarified and reinforced in the revised Discussion (page 16, lines 289–292) to improve clarity and alignment with the reviewer’s interpretation.

Finally, the language regarding oxygen therapy has been softened to remain consistent with the observational nature of the study (page 16, lines 282–286).

Comment 9: Incomplete reporting of key study limitations, including confounding by indication, selection bias, lack of cause-of-coma classification, and absence of validated severity scores (e.g., APACHE/SOFA).

Response: We thank the reviewer for this important comment.

The Limitations section has been revised to explicitly acknowledge the absence of validated severity scores (e.g., APACHE/SOFA), which were not available in the dataset, and the potential confounding by indication affecting interpretation of management-related variables. We also clarify that inclusion of ICU patients only may limit the generalizability of the findings (page 17, lines 313–322).

In addition, the classification of the cause of impaired consciousness was clinically assessed but not reported in the original manuscript. This has now been added to Table 1 in the revised version (page 10).

Comment 10: Overstatement in the conclusion regarding the effect of oxygen therapy on mortality, requiring revision to avoid causal implication and align with observational evidence.

Response: We thank the reviewer for this important comment. The Conclusion has been revised to avoid overstatement regarding the effect of oxygen therapy on mortality. The wording has been softened to reflect the observational nature of the study and to avoid causal interpretation, in line with the available evidence. The revised conclusion now emphasizes oxygen availability as a priority area for strengthening essential critical care rather than a direct mortality-reducing intervention.

Comment 11: Minor issue regarding interpretability of continuous variables in Table 2 due to reporting of hazard ratios per unit increase, with recommendation for rescaling (e.g., SBP per 10 mmHg).

Response: We thank the reviewer for this helpful comment. To ensure consistency across all analyses, continuous variables, including SBP and DBP, were retained in their original units in Table 2 (univariable Cox model). In the multivariable model (Table 3), SBP was the only blood pressure variable that remained independently and significantly associated with the outcome. For improved clinical interpretability, its effect was additionally rescaled in the Discussion to reflect a 10-mmHg increase by multiplying the regression coefficient (log-HR) by 10 before exponentiation, with the corresponding 95% CI recalculated on the log scale (page 15, lines 274–275). This rescaling was applied solely for interpretative purposes and did not modify the underlying statistical models.

Comment 12: Key revisions needed including clarification of cohort definition (GCS score 9–14), strengthening of the literature review with African ICU studies, cautious interpretation of management-related variables due to confounding by indication, expansion of study limitations, clarification of database creation, and improved contextualization of high ICU mortality.

Response: We thank the reviewer for this synthesis of key required revisions. The manuscript has been substantially revised accordingly. Cohort definition has been clarified, including justification of the inclusion of patients with GCS score 9–14. The literature review has been strengthened with additional African ICU studies to improve contextualization. Management-related variables are now interpreted cautiously, with explicit consideration of confounding by indication. The limitations section has been expanded to reflect key methodological constraints. The study database creation process has been clarified to improve transparency. Finally, the Discussion has been revised to better contextualize the high ICU mortality in relation to existing literature and limited ICU capacity.

---------------------------------------------------------------------------------------------------------------

Responses to Reviewer 2

Comment 1: Misalignment between the Introduction and the study focus, with excessive emphasis on critical care systems rather than on impaired consciousness as a clinical entity and its epidemiology.

Response: We thank the reviewer for this helpful comment. The Introduction has been revised to place greater emphasis on impaired consciousness as a clinical entity and on its epidemiology, in line with the title and objectives of the manuscript. The broader discussion of critical care systems has been streamlined and retained only where needed for context in resource-limited settings.

Comment 2: Need for clarification of the use of data from 2015–2017 and justification of the current relevance of the findings given potential changes in ICU capacity and practices over time.

Response: We thank the reviewer for this important comment. The use of data from 2015–2017 reflects the availability of an ICU database constructed from archived medical records, as this ICU does not routinely maintain a structured research registry. These findings should therefore be interpreted in the context of the limited critical care capacity typical of many low- and middle-income countries during the study period. Although the data are based on ICU admissions from 2015 to 2017, they remain an important source of information on ICU outcomes among adults with impaired consciousness in Benin. This study therefore contributes valuable evidence in a still under-described clinical setting, and this temporal limitation has been acknowledged in the revised manuscript.

Comment 3: Need for clarification of the definition of impaired consciousness, given the inclusion of a wide GCS range (3–14), introducing clinical heterogeneity, and recommendation to discuss this limitation and consider sensitivity analyses.

Response: We thank the reviewer for this thoughtful comment.

The study included all patients with a Glasgow Coma Scale (GCS) score of 3–14 at ICU admission to capture the full spectrum of impaired consciousness encountered in routine practice at this ICU. The different levels of impaired consciousness (mild, moderate and severe) are now clearly taken into account in Table 1 (page 10).

To address the concern about clinical heterogeneity, we performed subgroup

Attachment Submitted filename: Response_to_Reviewers.docx

# Editor report: Decision Letter 1

**DOI:** 10.1371/journal.pone.0350120.r003

**Authors:** Nik Hisamuddin Nik Ab. Rahman, Nik Hisamuddin Nik Ab. Rahman

11 May 2026

Survival and factors associated with intensive care unit mortality among adults with impaired consciousness in Benin, a low-resource setting: A retrospective cohort study

PONE-D-26-08886R1

Dear Dr. Sossou,

We’re pleased to inform you that your manuscript has been judged scientifically suitable for publication and will be formally accepted for publication once it meets all outstanding technical requirements.

Within one week, you’ll receive an e-mail detailing the required amendments. When these have been addressed, you’ll receive a formal acceptance letter and your manuscript will be scheduled for publication.

An invoice will be generated when your article is formally accepted. Please note, if your institution has a publishing partnership with PLOS and your article meets the relevant criteria, all or part of your publication costs will be covered. Please make sure your user information is up-to-date by logging into Editorial Manager at Editorial Manager® and clicking the ‘Update My Information' link at the top of the page. For questions related to billing, please contact billing support.

If your institution or institutions have a press office, please notify them about your upcoming paper to help maximize its impact. If they’ll be preparing press materials, please inform our press team as soon as possible -- no later than 48 hours after receiving the formal acceptance. Your manuscript will remain under strict press embargo until 2 pm Eastern Time on the date of publication. For more information, please contact onepress@plos.org.

Kind regards,

Nik Hisamuddin Nik Ab. Rahman

Academic Editor

PLOS One

Additional Editor Comments (optional):

Reviewers' comments:

# Editor report: Acceptance letter

**DOI:** 10.1371/journal.pone.0350120.r004

**Authors:** Nik Hisamuddin Nik Ab. Rahman, Nik Hisamuddin Nik Ab. Rahman

PONE-D-26-08886R1

PLOS One

Dear Dr. Sossou,

I'm pleased to inform you that your manuscript has been deemed suitable for publication in PLOS One. Congratulations! Your manuscript is now being handed over to our production team.

At this stage, our production department will prepare your paper for publication. This includes ensuring the following:

* All references, tables, and figures are properly cited

* All relevant supporting information is included in the manuscript submission,

* There are no issues that prevent the paper from being properly typeset

You will receive further instructions from the production team, including instructions on how to review your proof when it is ready. Please keep in mind that we are working through a large volume of accepted articles, so please give us a few days to review your paper and let you know the next and final steps.

Lastly, if your institution or institutions have a press office, please let them know about your upcoming paper now to help maximize its impact. If they'll be preparing press materials, please inform our press team within the next 48 hours. Your manuscript will remain under strict press embargo until 2 pm Eastern Time on the date of publication. For more information, please contact onepress@plos.org.

You will receive an invoice from PLOS for your publication fee after your manuscript has reached the completed accept phase. If you receive an email requesting payment before acceptance or for any other service, this may be a phishing scheme. Learn how to identify phishing emails and protect your accounts at https://explore.plos.org/phishing.

If we can help with anything else, please email us at customercare@plos.org.

Thank you for submitting your work to PLOS ONE and supporting open access.

Kind regards,

PLOS ONE Editorial Office Staff

on behalf of

Professor Dr Nik Hisamuddin Nik Ab. Rahman

Academic Editor

PLOS One
